# 75 years of dryland science: Trends and gaps in arid ecology literature

**DOI:** 10.1371/journal.pone.0175014

**Published:** 2017-04-06

**Authors:** Aaron C. Greenville, Chris R. Dickman, Glenda M. Wardle

**Affiliations:** 1 Desert Ecology Research Group, School of Life and Environmental Sciences, University of Sydney, Sydney, Australia; 2 National Environmental Science Programme Threatened Species Recovery Hub, University of Sydney, Sydney, Australia; 3 Long Term Ecological Research Network, Terrestrial Ecosystem Research Network, Sydney, Australia; University of Tasmania, AUSTRALIA

## Abstract

Growth in the publication of scientific articles is occurring at an exponential rate, prompting a growing need to synthesise information in a timely manner to combat urgent environmental problems and guide future research. Here, we undertake a topic analysis of dryland literature over the last 75 years (8218 articles) to identify areas in arid ecology that are well studied and topics that are emerging. Four topics—wetlands, mammal ecology, litter decomposition and spatial modelling, were identified as ‘hot topics’ that showed higher than average growth in publications from 1940 to 2015. Five topics—remote sensing, climate, habitat and spatial, agriculture and soils-microbes, were identified as ‘cold topics’, with lower than average growth over the survey period, but higher than average numbers of publications. Topics in arid ecology clustered into seven broad groups on word-based similarity. These groups ranged from mammal ecology and population genetics, broad-scale management and ecosystem modelling, plant ecology, agriculture and ecophysiology, to populations and paleoclimate. These patterns may reflect trends in the field of ecology more broadly. We also identified two broad research gaps in arid ecology: population genetics, and habitat and spatial research. Collaborations between population genetics and ecologists and investigations of ecological processes across spatial scales would contribute profitably to the advancement of arid ecology and to ecology more broadly.

## Introduction

A goal of each discipline in science is to integrate knowledge to achieve progressively deeper understanding of the natural world. However, with the exponential growth in scientific publications [1, 2], there is a growing need to synthesise information in a timely manner. We have now entered the era of ‘big data’ and, with the recent development of new statistical techniques, researchers can summarise information and identify past and future trends in research topics with increasing ease. The diminishing worldwide allocation of resources for natural science [3] also presents an increasing imperative to identify 'important' topics that bear on fundamental understanding of the natural world.

Identifying topics and research directions can provide insights into how a discipline is changing over time [4]. For example, bibliometric reviews have explored research trends in specific fields, such as how research in urban ecology aligns with professional society statements of research goals [5], how the ecological surrogate and indicator literature informs conservation science [6] and topics studied within specific journals [7]. Bibliometric reviews have also been used to track topics in the field of ecology as a whole [4, 8, 9]. For example, Carmel *et al*. (9) showed that, from a survey of 750 articles, 70% of articles studied single species, whereas ecosystem and community studies represented 25%.

Past attempts to define key topic areas often have been made arbitrarily at the national level (e.g. Australia's science and research priorities), via expert panels (e.g. National Science Foundation in the USA), or more broadly through consultation with the global corpus of ecologists, such as by horizon scanning [10]. However, these approaches are prone to problems such as sample bias and limited sample size. A novel alternative method is to use published literature as a guide to identify which topics are being pursued most avidly (hot topics), which are being ignored (cold topics), which are coalescing, and those with key gaps. Topic analysis, such as Latent Dirichlet Allocation [11], has become available very recently; with the advent of large literature databases that contain decades of primary literature across all science areas, and tools that allow extraction of such 'big data' information, researchers can now for the first time survey potentially all relevant literature in a research area.

In this study we undertake a topic analysis to identify areas that are studied in ecology and how the growth in topics has changed over the past 75 years. Given the broad scope of this inquiry, we review in detail a particular discipline—arid ecology—and use it as a case study to interrogate an entire corpus of literature. We then identify areas that are well studied and where generality is likely, as well as emerging or neglected areas that would be fruitful to pursue. Arid ecology was selected because of its critical role in maintaining biodiversity, human livelihoods and environmental integrity, and in reducing desertification, which is a major driver of human conflict [12, 13]. We take our review from 1940. Scientific literature records go back reliably to this period, in part because of the flowering of science since World War II, allowing time for topics to be developed. We identify hot and cold topics in the literature, discover if similar topics cluster together, and identify research gaps.

## Materials and methods

### Literature review and corpus

To review the literature on arid ecology we searched the ISI Web of Science with the following keywords: arid *and* ecology *and* terrestrial. We sorted articles by relevance and exported all articles with their abstracts from 1940 up to the end of 2015. Our search returned 9175 articles from this period. We removed articles with abstracts that did not share common words, resulting in 8218 articles [14] and 49 913 shared words. These final articles made up the corpus, which was then converted into a document term matrix. To remove very common words, stop words (for example, *the* or *and*) were filtered out and minimum word length was set to three characters. All numbers and punctuation were removed, and 'stemming' (words reduced to their base or root form) was used to help identify important topic words (see below). The document term matrix was generated using the topic models 0.2–2 package [11] in the R Statistical program [15].

### Topic identification

We used Latent Dirichlet Allocation (LDA) to identify common topics reported in the literature. LDA identifies sets of words that occur together with unusual frequency, thus allowing topics to be defined [6]. Multiple topics can occur in any article, and LDA weights topics within an article and the words that define a topic [11]. We set the number of topics to 25, which adequately represented variation in the data, captured the complexity of topics in the literature, but was not so high as to hamper interpretation of patterns [6]. We named each topic based on assessment of the 20 highest weighted words for that topic and expert knowledge of the literature (S1 Table). LDA was performed using the topic models 0.2–2 package [11] in the R Statistical program [15].

### Topic popularity, growth and hot topics

To determine topic popularity, the number of published articles per topic over time (years) was analysed using a Poisson Generalised Linear Mixed Model (GLMM) with a random intercept (i.e. mean number of publications) and slope (i.e. rate of change in number of publications across years). To aid in interpretation, data were centred by subtracting the mean and dividing by the standard deviation (z-scored) as described in Westgate *et al*. (6). Topics with positive random intercepts can be interpreted as having higher than average numbers of articles written about them and topics with positive random slopes as having higher than average growth in publications in the time period (1940-2015) [6, 7]. GLMM was run using the lme4 1.1–10 package [16] in the R Statistical program [15].

### Topic similarity and research gaps

LDA produces a matrix of the weight of each word (n = 49 913) within each topic (n = 25) [6, 11]. To investigate topic similarity, we calculated the Euclidean distance between each pair of topics using a matrix of the weight of each word within each topic (‘word’ distance matrix). We then plotted topic similarity as a dendrogram using APE package 3.4 [17] in the R Statistical program [15]. In addition to this matrix, LDA produces a matrix of the weight of each topic (n = 25) within each article (n = 8218) [6, 11]. We transposed this matrix so topics were rows and article weights were columns, and then calculated the Euclidean distance between each pair of topics using the matrix of topics and article weights (‘article’ distance matrix). To compare the difference in topics based on words versus articles, we calculated the product of the ‘word’ and ‘article’ distance matrices (‘gap’ distance matrix), after each had been scaled between zero and one [see 6].

We investigated research gaps between topics by plotting the dissimilarity metric from the ‘gap’ distance matrix. The greater the metric, the higher the dissimilarity between topics (topics that both contain different sets of words and topics that rarely co-occur in the same article [6]).

## Results

### Topic identification, popularity, growth and hot topics

**Fig 1:**
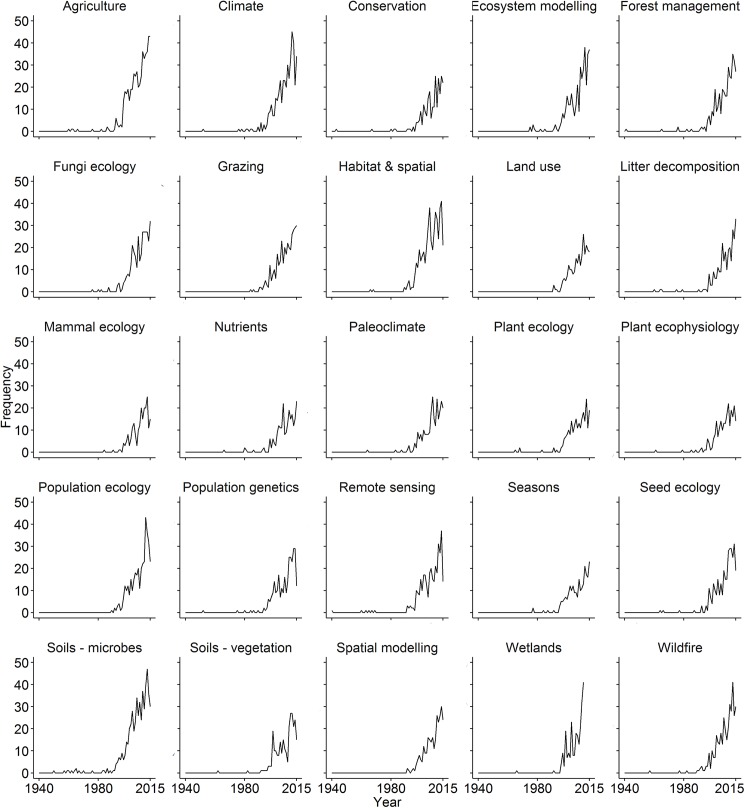
Growth in research articles per topic. The frequency per year of research articles published for each topic area in the global dryland literature from 1940 to 2015. Topics were identified by Latent Dirichlet Allocation using abstracts from 8218 articles.

**Fig 2:**
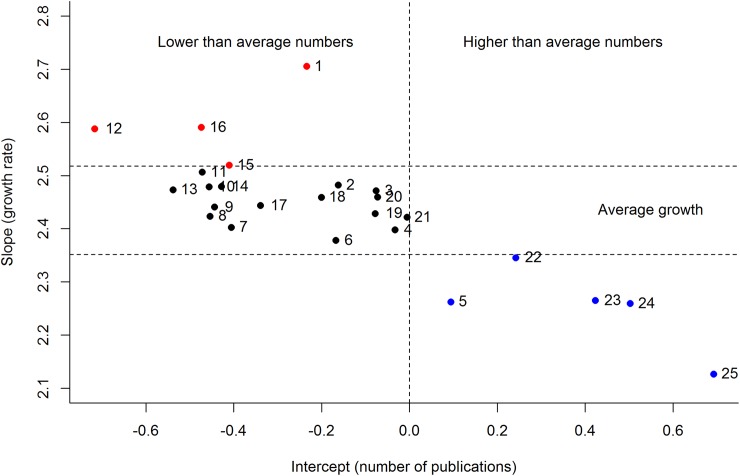
Hot topics in global dryland research. Identified hot (red circles) and cold (blue circles) topics in global dryland literature from Poisson Generalised Linear Mixed Model slopes and intercepts. Topics with positive random intercepts can be interpreted as having higher than average numbers of articles written about them, and topics with random slopes greater than the fixed-effect mean (± 95% confidence interval) have higher than average growth in publications in the time period analysed (1940-2015). Topics with average growth over the study period are shown with black circles. See Fig 3 and S1 Table for identification of topic numbers.

From 25 topic areas and using 8218 articles in the literature on arid ecology, Latent Dirichlet Allocation identified topics ranging from soils, litter decomposition, plant and mammal ecology to ecosystem and population-scale ecology and biology (S1 Table). The number of publications in arid ecology has grown exponentially since 1940 across all topic areas (GLMM: fixed effect estimate = 2.4, z-value = 57.4, *P* < 0.01; Fig 1), similar to global trends in research output [1]. Soils and agriculture were the initial topics of interest, but after 1980 a plethora of topics started to be investigated (Fig 1). Two topics, mammal ecology and wildfire, contained ‘Australia’ in the top 20 words (S1 Table), the latter being the only regional area or country to be thus identified. Four topics—wetlands, mammal ecology, litter decomposition and spatial modelling, were identified as ‘hot topics’ receiving higher than average growth in publications from 1940 to 2015 (Fig 2). However, all ‘hot topics’ had lower than average numbers of publications across the study period. Five topics—remote sensing, climate, habitat and spatial, agriculture and soils-microbes, were identified as ‘cold topics’, with lower than average growth, but higher than average numbers of publications from 1940 to 2015 (Fig 2). Sixteen of the 25 topics showed average growth in publications from 1940 to 2015 (Fig 2).

### Topic similarity and research gaps

**Fig 3:**
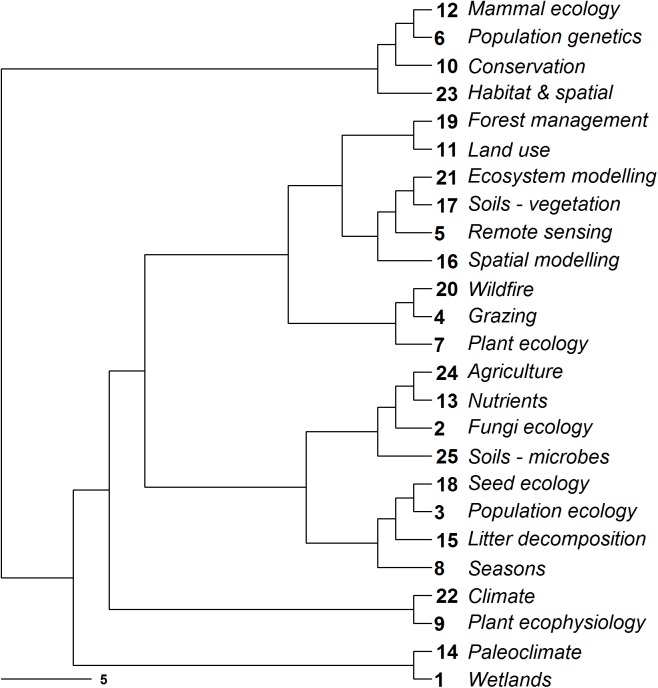
Topic similarity in global dryland research. Topic similarity in global dryland literature calculated using the Euclidean distance between each pair of topics using a Latent Dirichlet Allocation matrix of the weight of each word within each topic.

The 25 topics clustered into seven broad groups on word-based similarity (Fig 3). The first group represented work on mammals, population genetics, conservation, and habitat and spatial research. The second group consisted of broad-scale management and ecosystem modelling (dryland forest management, land use, ecosystem modelling, soils-vegetation, remote sensing and spatial modelling). The third group focused on plant ecology and processes such as grazing and wildfire, and the fourth on topics related to agriculture (nutrients, ecology of fungi and soils–microbes). The fifth group focused on topics associated with processes related to populations (seed ecology, population ecology, litter decomposition and seasons), and the sixth was associated with ecophysiology (climate and plant ecophysiology). Lastly, paleoclimate and wetlands topics clustered together (Fig 3).

**Fig 4:**
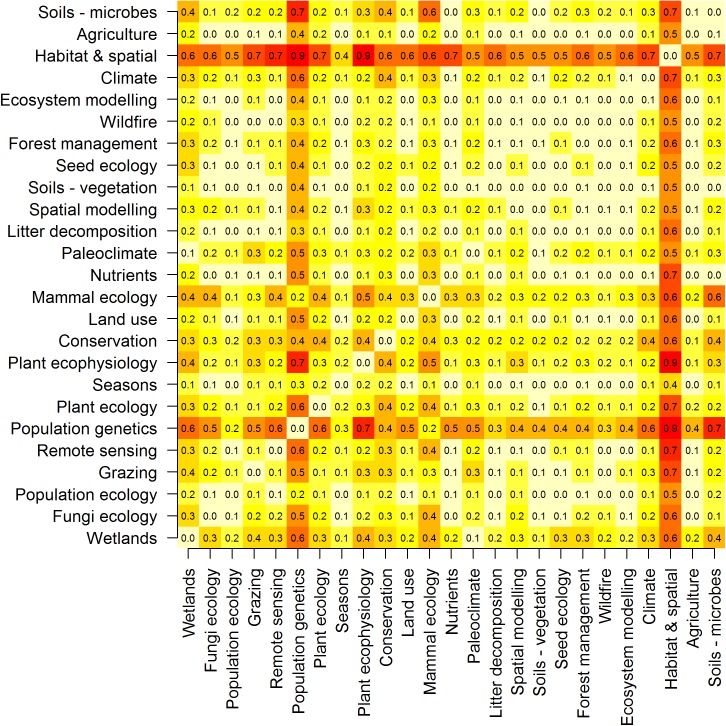
Research gaps in global dryland literature. Research gap distance matrix heat-map (red = high, clear = low) on global dryland literature. The greater the metric the higher the dissimilarity between topics (i.e. topics that both contain different sets of words and topics that rarely co-occur in the same article).

Analysis of research gaps indicates that population genetics and habitat and spatial research show the largest degree of separation from the other 23 topics (Fig 4). The topics population genetics and population ecology, especially mammal ecology, have been jointly investigated, but future research could target gaps between the other topics identified (Fig 4).

## Discussion

Over the past 75 years, topics studied in dryland or arid ecology have ranged from soils, litter decomposition, plant and mammal ecology, to population and ecosystem-scale ecology. The four identified ‘hot topics’ had lower than average numbers of publications but relatively high rates of growth, while the five ‘cold topics’ had higher than average numbers of publications but slow growth, suggesting that topic areas diffuse through the literature over time. Some cold topics, such as agriculture and soils, were an early focus of research in arid ecology, allowing more time to diffuse through the literature. There was no evidence that hot or cold topics clustered together, suggesting that the seven broad areas of research identified in the ordination analysis remained equally studied, and that topics within them have shifted in popularity. Contrary to broader research trends in ecology, climate was identified as a cold topic. Climate is currently emerging in ecology [4, 9], but is already a well-established topic within arid ecology, presumably due to its critical role in driving arid systems [18]. Thus, studies both past and present in arid ecology may contribute greatly to our understanding of changes in extreme weather events and the evolutionary responses of biota and ecological systems under future climate change.

The 25 identified topics in arid ecology clustered into seven broad groups on word-based similarity. These groups ranged from mammal ecology and population genetics, broad-scale management and ecosystem modelling, plant ecology, agriculture and ecophysiology, to populations and paleoclimate. These trends may reflect the field of ecology more broadly. For example, we found that mammals grouped with population genetics and plant ecology with broader ecosystem processes, such as wildfire and grazing. By analysing keyword associations in ecology journals, Budilova *et al*. (8) found the keyword ‘mammals’ to be associated with ‘population dynamics’ and ‘plants’ with ‘ecosystems’. Our finding that ‘Australia’ was among the topic words for ‘mammal ecology’ and ‘wildfire’ similarly reflects recent burgeoning of interest in these topics in the continent's desert regions [19, 20].

This study identified two broad research gaps within arid ecology. Population genetics and habitat and spatial research showed the largest degree of separation from the other 23 topics. Genetics is a hot topic in ecology more generally [4], but within arid ecology it is still associated mainly with studies of mammals. Within the literature on ecological surrogates and indicators, genetics also showed a large degree of separation from the other topics identified [6]. Even though genetics has been identified as one of the new frontiers of ecology [21], the separation of genetics from other topics in ecology may be because of the specialist methods and training that are required. We suggest that further efforts and collaborations could be targeted at gaps between population genetics and plant ecology or microbiology. In addition, research gaps exist between population genetics and processes that may influence species populations, such as grazing, climate and habitat use or spatial dynamics. Further collaborations targeting these research gaps will advance our understanding of ecology and evolutionary processes, as shown by recent works that have synthesised phylogenetic, geographic and paleo-environmental data to track the assembly of arid biotas [22, 23].

Habitat and spatial topics showed large separation from the other topics identified, including remote sensing, suggesting that within arid ecology there should be a research focus on linking the various scales at which ecology operates. Linking global environmental change to its effects at smaller scales, including effects on individual species, is a fundamental challenge for modern ecology [24]. However, recent advances in technology and data analysis, such as high-resolution satellite imagery, drones, remote cameras and open-source hardware, now allow multi-scale methods to be deployed to better understand ecological processes that operate across ecosystems [25, 26]. We suggest that new and exciting collaborations could be developed across these research topics.

Topic analysis can have limitations. For example, by only analysing the available literature, novel topics or topics yet to emerge are hard to identify [6]. Thus, studies such as ours can only identify gaps between existing topics. Despite this, topic analysis provides a valuable method to guide researchers as to where they can look for novel or emerging topics as well as research gaps where they can increase their research efforts. The use of article keywords or abstracts also may limit the information available to text summary methods, and thus fail to capture some topics or the miss the indented meaning behind word usage [6, 27]. For example, simplifying articles based on common words may miss the meaning or conclusions of a study, as the words may be used out of context [28]. As computing power increases and more full-text open-access articles become available, these issues will reduce. In this study, we used article abstracts to increase the information available to text summary methods, rather than article keywords, and surveyed the entire corpus of literature in arid ecology to increase our sample size. Surveying the entire corpus of 75 years of abstracts across a research field thus provided a useful means to summarise knowledge and identify research directions.

Our topic analysis was successful in identifying past and existing areas of endeavour in the arid ecological literature. However, research gaps also are evident, particularly in population genetics and habitat and spatial research, suggesting that similar gaps within ecology may apply more broadly within arid ecology. New and exciting collaborations between population genetics and plant ecology, or investigations of ecological processes across spatial scales, are among several topic areas that have the potential to contribute much to the advancement of arid ecology and to ecology more broadly.

## Supporting information

S1 Table Topic classification. (DOCX)
