# Supplementary material for: 75 years of dryland science: Trends and gaps in arid ecology literature
Source: PLoS One. 2017 Apr 6;12(4):e0175014. doi: 10.1371/journal.pone.0175014 (PMC5383157; doi:10.1371/journal.pone.0175014)
Supplement: S1 Table — (DOCX) [file pone.0175014.s001.docx]

**Dry science: arid ecology has matured as a science**

**Supplementary Section: Topic classification**

**Table S1:** Topic classification from the top 20 topic words identified from Latent Dirichlet Allocation, including the theory and domain that each topic contributed to.

| Topic number | Topic name | Theory/domain | Topic words |
| --- | --- | --- | --- |
| 1 | Wetlands | Models/Ecosystems | flood, deposit, late, wetland, climat, fossil, floodplain, dure, arid, terrestri, river, basin, earli, fauna, assemblag, format, record, indic, suggest, stream |
| 2 | Fungi ecology | Models/Organismal systems | root, plant, growth, fungi, speci, soil, mycorrhiz, amf, isol, host, salin, arid, fungal, salt, stress, studi, differ, semiarid, show, colon |
| 3 | Population ecology | Constitutive theory/Population systems | popul, sand, speci, differ, dune, reproduct, plant, rate, adapt, growth, surviv, condit, size, environment, higher, trait, habitat, high, signific, height |
| 4 | Grazing | Models/Management & conservation | graze, speci, plant, cover, site, rich, divers, veget, effect, differ, abund, increas, shrub, composit, grass, communiti, signific, species, densiti, grazing |
| 5 | Remote sensing | Models/Ecosystem | veget, tree, use, cover, data, ndvi, canopi, area, index, differ, biomass, imag, studi, semiarid, estim, measur, indic, spectral, remot, satellit |
| 6 | Population genetics | Constitutive theory/ Population systems | popul, speci, genet, size, breed, habitat, femal, arid, rang, group, use, male, differ, nest, within, ecolog, two, studi, species, suggest |
| 7 | Plant ecology | Models/Ecosystem‑Organismal systems | plant, communiti, effect, speci, interact, chang, pattern, respons, function, ecosystem, arid, import, competit, increas, structur, may, differ, result, spatial, relat |
| 8 | Seasons | Population systems‑ Organismal systems | season, dure, summer, nest, winter, spring, activ, ant, rabbit, termit, autumn, popul, densiti, year, abund, dri, area, may, studi, month |
| 9 | Plant ecophysiology | Models/Organismal systems | water, leaf, plant, soil, speci, use, delta, drought, differ, increas, root, tree, groundwat, content, high, growth, relat, rate, potenti, dure |
| 10 | Conservation | Management & conservation | arid, ecolog, region, conserv, area, speci, research, habitat, develop, natur, ecosystem, includ, protect, manag, mani, popul, human, land, use, desert |
| 11 | Land use | Management & conservation | veget, land, soil, area, china, degrad, grassland, restor, region, use, result, studi, chang, cover, arid, forest, plantat, water, increas, show |
| 12 | Mammal ecology | Constitutive theory‑Models/Population systems‑ Organismal systems | speci, habitat, mammal, diet, food, prey, predat, use, small, rodent, arid, studi, forag, dure, popul, australia, abund, high, activ, species |
| 13 | Nutrients | Constitutive theory‑Models/Ecosystems | soil, carbon, increas, biomass, grassland, nitrogen, ecosystem, effect, product, soc, signific, semiarid, rate, total, organ, aboveground, net, nutrient, result, year |
| 14 | Paleoclimate | Models/Ecosystems | pollen, lake, dure, climat, chang, sediment, record, arid, terrestri, flower, veget, indic, region, pollin, similar, period, marin, isotop, increas, cal |
| 15 | Litter decomposition | Models/Ecosystems | litter, decomposit, leaf, season, rate, nutrient, dri, dure, forag, concentr, differ, speci, leav, plant, qualiti, goat, mass, semiarid, tree, loss |
| 16 | Spatial Modelling | Models/Ecosystems | use, model, data, distribut, spatial, studi, variabl, area, veget, method, analysi, sampl, pattern, environment, landscap, relationship, estim, map, base, differ |
| 17 | Soils - vegetation | Models/Ecosystems | soil, patch, veget, spatial, shrub, cover, pattern, surfac, semiarid, differ, area, landscap, scale, runoff, eros, water, heterogen, moistur, effect, bare |
| 18 | Seed ecology | Constitutive theory‑Models/Population systems‑ Organismal systems | seed, seedl, speci, plant, germin, dispers, nativ, invas, establish, effect, bank, surviv, species, soil, recruit, emerg, arid, differ, studi, may |
| 19 | Forest management | Management & conservation | forest, manag, chang, tree, climat, use, area, rangeland, increas, popul, land, impact, model, livestock, semiarid, system, effect, rang, savanna, will |
| 20 | Wildfire | Models/Ecosystems | fire, grass, year, burn, woodland, increas, cover, rainfal, area, grassland, veget, semiarid, time, speci, savanna, mortal, tree, effect, australia, chang |
| 21 | Ecosystem modelling | Models/Ecosystems | model, ecosystem, water, use, simul, estim, flux, measur, npp, product, climat, semiarid, surfac, data, predict, chang, result, energi, veget, hydrolog |
| 22 | Climate | Models/Ecosystems | temperatur, climat, precipit, season, annual, rainfal, chang, increas, dure, region, degre, year, mean, veget, variat, differ, arid, period, desert, temperature |
| 23 | Habitat & spatial | Models/Ecosystems | speci, habitat, species, region, divers, area, rich, arid, forest, distribut, communiti, taxa, plant, bird, ant, abund, studi, desert, found, record |
| 24 | Agriculture | Management & conservation | crop, irrig, yield, plant, soil, water, use, agricultur, product, increas, treatment, field, system, high, signific, effect, higher, wheat, studi, applic |
| 25 | Soils – microbes | Models/Ecosystems | soil, crust, organ, microbi, communiti, signific, content, differ, biolog, soils, desert, activ, surfac, matter, studi, semiarid, arid, sampl, higher, moistur |
